# Promoting Vaccination in the Post-COVID-19 Era

**DOI:** 10.3390/vaccines14010086

**Published:** 2026-01-15

**Authors:** Zoi Tsimtsiou

**Affiliations:** Department of Hygiene, Social-Preventive Medicine and Medical Statistics, School of Medicine, Aristotle University of Thessaloniki, 54124 Thessaloniki, Greece; ztsimtsiou@auth.gr

## 1. Background

During the COVID-19 pandemic, substantial changes occurred that may have either facilitated or hindered efforts to promote vaccination. While the introduction of COVID-19 vaccines has given rise to new challenges related to vaccine acceptance, the pandemic has also represented a unique opportunity to increase public awareness of prevention practices, including immunization [1]. In developing effective interventions to improve vaccination coverage in the post-pandemic context, it is essential to assess individuals’ beliefs and attitudes, together with social influences, practical barriers, and evidence-based strategies derived from lessons learned that may affect motivation and the ability to be vaccinated. To provide a comprehensive understanding of the multifaceted determinants that must be addressed to effectively tackle suboptimal vaccination coverage in the post-pandemic context, nine articles were selected through a rigorous peer-review process for inclusion in this Special Issue.

## 2. Collection of Special Issue Articles

The article by Feijó et al. [2] presents the results of a prospective study that investigates the interplay between vaccine confidence, sociodemographic variables and vaccine uptake among 237 undergraduate medical students in Brazil, by analyzing vaccination records and data deriving from questionnaires. The results revealed suboptimal vaccination rates despite the high reported levels of vaccine confidence. Higher immunization rates were positively associated with advanced clinical training and specific socioeconomic factors, namely higher family income and private secondary education. The study emphasized the need to review policies, where appropriate, to include medical students in priority groups within National Immunization Programs, as well as to implement relevant educational interventions during undergraduate training.

The article by Huang et al. [3] uses data collected with questionnaires from 232 caregivers from outpatient clinics in Taiwan to identify significant predictors of their intention to vaccinate their elderly family members with Major Neurocognitive Disorders against COVID-19. The results reveal that caregivers’ perceived familial support for vaccination, the perceived value of vaccination, and autonomy to vaccinate elder family members were positively correlated with their intention to vaccinate them. Conversely, caregivers showed a lower intention to vaccinate them as the age of the elderly family member increased. The findings suggest that healthcare interventions should focus on enhancing caregivers’ motors of vaccination, such as value, positive impacts, and autonomy, and engaging the broader family unit to improve vaccination rates among this high-risk population.

The article by Ferrari et al. [4] evaluates the seroprevalence of mumps-specific IgG antibodies in a cohort of 468 healthcare workers in Italy. The serological study revealed that 8.3% were unprotected against mumps, falling below the 95% threshold required for herd immunity. The findings indicate that a positive vaccination history is an unreliable indicator of actual immunity, as antibody levels often wane significantly over time or fail to reach protective thresholds after the standard two-dose schedule. This article suggests the necessity of serological screening for mumps to identify and revaccinate non-immune healthcare staff prior to employment, regardless of their vaccination history.

The article by Papagiannis et al. [5] evaluates immunity levels against diphtheria and tetanus among 1201 Greek adults using geographically stratified sampling based on regional units by considering variables such as age group (30–80+ years old) and sex. The findings revealed that the overall seropositivity rate for diphtheria IgG antibodies was 31.5%, while for tetanus it was 59.5%. Younger age groups were associated with tetanus seropositivity, while male sex and an age over 80 years old were associated with diphtheria seropositivity. Additionally substantial regional variations were observed. This nationwide seroprevalence study emphasizes the need for public health and primary care-driven education on adult vaccinations, with emphasis on repeated booster shots throughout adulthood to sustain community protection and maintain herd immunity.

The article by Avramidis et al. [6] investigates attitudes regarding adult vaccination in the aftermath of the pandemic, as well as the vaccination coverage against five communicable diseases and the predictors of their uptake in 395 adults visiting pharmacies. The findings revealed high coverage for COVID-19 and seasonal influenza among high-risk groups, while immunization rates for pneumococcal disease, herpes zoster, and tetanus remained suboptimal. A doctor’s recommendation was the strongest predictor of uptake for each vaccine (OR range 11.3–37.7, *p* < 0.001), followed by a pharmacist’s recommendation (OR range 4–19.5, *p* < 0.05), with the exception of COVID-19 vaccination, which was uniquely predicted by individuals’ belief in the overall value of adult immunization. Qualitative analysis highlighted several universal barriers to vaccination, including negligence, perceived low susceptibility to disease, and insufficient information, though safety concerns and distrust in authorities were reported exclusively regarding the COVID-19 vaccine. The findings highlight the crucial role of health professionals in strongly recommending adult vaccination and underscore the need to design public interventions that enhance trust in adult immunization in the aftermath of the pandemic.

The article by Damnjanović et al. [7] uses a cross-sectional, online study design to investigate the complex interplay between social roles and psychological factors in determining vaccination intention among 745 participants in Serbia, including healthcare providers, parents, and laypeople. Findings revealed that negative vaccine attitudes represent a primary barrier to vaccination intent across all demographic cohorts. A consistent “attitudinal core” was identified as the basis for positive vaccine intention, characterized by high levels of institutional trust, perceived scientific consensus regarding safety and efficacy, and a subjective experience of personal freedom. Conversely, vaccine hesitancy was significantly correlated with vaccination-specific conspiracy beliefs, high reliance on social media for information, and the psychological burden of choice overload. Notably, promotive factors were group-specific: parental intent was driven by institutional trust and perceived consensus, and healthcare provider intent was motivated by the experience of freedom, while no unique promotive factors were isolated for the lay population. Ultimately, the study suggests that while there are common protective factors, immunization interventions must be tailored to the specific social roles of individuals to be effective in a post-pandemic landscape.

The article by Pennisi et al. [8] synthesizes critical insights from the pandemic, underscoring the imperative for adaptable and resilient public health infrastructures capable of rapid mobilization during emerging health crises. This narrative review indicates that robust vaccination uptake is contingent upon resilient supply chain logistics, transparent communication frameworks, proactive community engagement, and equitable resource distribution. Furthermore, the need for multi-component strategic approaches that integrate cross-sectoral partnerships with local stakeholders, precision-tailored messaging, and the deployment of digital health tools to mitigate vaccine hesitancy and bolster public confidence is emphasized. This review suggests that the post-pandemic era provides an opportunity to strengthen global cooperation and public health resilience by applying these evidence-based insights to future vaccination campaigns.

The article by Al hashimi et al. [9] evaluates the optimal timing and integration of COVID-19 vaccinations in Canada, providing a comparative analysis with other G7 nations to inform national immunization policies. This systematic review indicates a growing international consensus toward integrating COVID-19 vaccinations with seasonal influenza campaigns, particularly to protect high-risk populations and maximize public health impact. Evidence suggests that the co-administration of COVID-19 and influenza vaccines is safe, maintains immunogenicity, and may even enhance spike-specific antibody responses while improving logistical efficiency and public acceptance. Furthermore, timely booster doses—particularly when administered at shorter intervals for older or immunocompromised individuals—significantly reduce infection rates, hospitalizations, and mortality by addressing the challenge of waning immunity. This systematic review suggests the transition toward integrated, seasonally aligned immunization programs supported by prospective studies to refine vaccination schedules and strengthen preparedness for future respiratory outbreaks.

The article by Dadari et al. [10] provides a comprehensive review of the developmental trajectory and operational insights derived from the global guidance framework development, jointly developed by WHO/UNICEF, during the acute phase of the COVID-19 pandemic. The guidance proposed a four-steps implementation model for the integration of COVID-19 vaccination, while identifying critical investment priorities for low- and middle-income countries. The project report demonstrates that guidance documents can be rapidly synthesized and deployed despite high-uncertainty environments. Multisectoral stakeholder collaboration, within and beyond immunization, is a potent catalyst for accelerating the integration of essential health services, essential for ensuring systemic preparedness against future pandemic threats.

## 3. Discussion

The articles included in this Special Issue collectively provide valuable insights into key priorities for promoting vaccination in the post-pandemic context. The findings underscore the importance of strengthening health professionals’ education beginning at the undergraduate level [11], not only to improve their own vaccination uptake, but also to enhance their knowledge and communication skills, which are essential for delivering strong and credible vaccination recommendations [12]. In parallel, health professionals can play an important role in engaging the broader family unit, where appropriate, and empower caregivers by increasing autonomy and reinforcing the perceived value of vaccination, particularly for high-risk populations.

Maintaining community protection requires a sustained focus on lifelong immunization, supported by increasing health literacy on booster doses during adulthood and by using serological screening to identify individuals who may require revaccination, irrespective of their vaccination history, especially among health workers. Public health strategies should also consider the needs of population groups with specific social roles, addressing psychological determinants such as institutional trust and perceived consensus regarding vaccine safety, while actively mitigating the influence of misinformation. Furthermore, the integration of COVID-19 vaccination into broader health system frameworks and seasonal influenza campaigns, supported by resilient supply chains and multi-component implementation strategies, has emerged as a critical factor for long-term public health resilience. Drawing on the lessons learned from the COVID-19 pandemic, the post-pandemic period represents an important opportunity to strengthen global cooperation and public health preparedness by translating these evidence-based insights into future vaccination-promotion strategies.
